# Acceptability and practicality of pGALS musculoskeletal screening tool in Malawian children

**DOI:** 10.1186/1546-0096-9-S1-P212

**Published:** 2011-09-14

**Authors:** E Smith, He Foster

**Affiliations:** 1 Royal Victoria Infirmary Hospital, Newcastle Upon Tyne, England, UK; 2 Musculoskeletal Research Group, Institute Cellular Medicine, The Medical School, Newcastle Upon Tyne, England, UK

## Background

Studies of rheumatic disease in children in Africa are lacking. The pGALS (paediatric Gait, Arms, Legs, Spine) Musculoskeletal (MSK) screen is validated in school aged English speaking children and shown to be practical and useful in acute paediatric practice.

## Aims

To evaluate the validity and practicality of pGALS in Malawian children in an acute hospital setting.

## Methods

Over a 2 day period, school-aged in-patients and children presenting to the Queen Elizabeth Hospital Blantyre, Malawi were consented to participate using a translator. Practicality (time taken, degree of completion) and patient / parent assessed acceptability (time take, discomfort) were assessed using a ‘smiley face’ visual analogue scale. The study had ethical approval.

## Results

Fifty-one children (median age 8 years); 23/51 (45%) assessed in emergency department, remainder assessed as in-patients; many (50%) had infection related diagnoses. Practicality of pGALS was good (median time taken to complete pGALS - 4 minutes (range 1.8-7.4), completed in 47/51 children (92%) – reason for non-completion; child being fractious (n=1), trauma related pain (n=1), limb fracture (n=1). Acceptability was high: 98% parents considered time taken to be acceptable, 84% of children deemed little / no discomfort. Abnormalities were found 21/51 (41%), including hypermobility, trauma / infection related, spinal deformity (Tuberculosis).

## Conclusions

The pGALS MSK screen was practical, acceptable and informative in this acute setting despite a language barrier. Many children were unwell with infection related problems but pGALS was completed in most; abnormal findings were common and the need to be interpreted within the clinical context was highlighted.

